# Molecular mechanisms involved in somatostatin receptor regulation in corticotroph tumors: the role of cytoskeleton and USP8 mutations

**DOI:** 10.1530/EO-22-0042

**Published:** 2022-03-23

**Authors:** Erika Peverelli, Donatella Treppiedi, Giovanna Mantovani

**Affiliations:** 1 Department of Clinical Sciences and Community Health, University of Milan, Milan, Italy; 2 Fondazione IRCCS Ca’ Granda Ospedale Maggiore Policlinico, Endocrinology Unit, Milan, Italy

**Keywords:** somatostatin receptors, filamin A, USP8, ACTH-secreting PitNET

## Abstract

Adrenocorticotropic hormone (ACTH)-secreting pituitary tumors mainly express somatostatin receptor 5 (SSTR5) since SSTR2 is downregulated by the elevated levels of glucocorticoids that characterize patients with Cushing’s disease (CD). SSTR5 is the molecular target of pasireotide, the only approved pituitary tumor-targeted drug for the treatment of CD. However, the molecular mechanisms that regulate SSTR5 are still poorly investigated. This review summarizes the experimental evidence supporting the role of the cytoskeleton actin-binding protein filamin A (FLNA) in the regulation of SSTR5 expression and signal transduction in corticotroph tumors. Moreover, the correlations between the presence of somatic USP8 mutations and the expression of SSTR5 will be reviewed. An involvement of glucocorticoid-mediated β-arrestins modulation in regulating SSTRs expression and function in ACTH-secreting tumors will also be discussed.

## Introduction

Adrenocorticotropic hormone (ACTH)-secreting pituitary neuroendocrine tumors (PitNETs) are the cause of Cushing’s disease (CD), a rare and potentially fatal systemic condition due to chronic exposure to cortisol, that results in many severe clinical manifestations such as cardiovascular disease, diabetes, hypertension, osteoporosis, infections, and thromboembolic events (Pivonello *et al.* 2020).

The first-line treatment for CD is pituitary surgery via transphenoidal route, that is followed by remission in about 70–90% of patients, with a 15–25% of recurrence risk and a 20–30% persistence risk (Pivonello *et al.* 2020). Thus, a consistent subgroup of patients requires second-line therapies. Among the available pharmacological options, the somatostatin receptor (SSTR) ligand pasireotide is the only approved pituitary tumor-targeted drug for the treatment of CD in adult patients (Colao *et al.* 2012).

SSTR family includes five different receptor subtypes (SSTR1–5). While the ﬁrst-generation somatostin analogs (SSAs), octreotide and lanreotide, have a high binding affinity for SSTR2 and, to a lesser extent, to SSTR5 and SSTR3, the second-generation SSA pasireotide is a multi-receptor targeted SSTR ligand that binds with higher affinity to SSTR5 (40-fold), SSTR1 (30-fold), SSTR3 (5-fold), when compared with octreotide (Bruns *et al.* 2002). However, pasireotide has a lower affinity than octreotide for SSTR2 (Bruns *et al.* 2002), in agreement with the higher doses required in the treatment of acromegaly.

The subtype of SSTR mainly expressed in ACTH-secreting pituitary tumors is SSTR5, whereas SSTR2 is expressed at lower levels (Hofland *et al.* 2005, van der Hoek *et al.* 2005, Batista *et al.* 2006, Ibáñez-Costa *et al.* 2016). Indeed, glucocorticoids have been shown to induce a significant downregulation of SSTR2, but not SSTR5, in corticotroph cells. A cortisol-induced downregulation of SS-binding sites was firstly described in 1982, although the SSTR subtype has not been characterized (Schonbrunn 1982). The involvement of SSTR2 has been hypothesized based on the observation that glucocorticoids reduced the antisecretory effects of octreotide in primary cultures of human corticotroph tumors (Stalla *et al.* 1994). Contrary to octreotide, pasireotide inhibitory effects on ACTH secretion were not affected by dexamethasone pretreatment in primary cultured tumoral corticotrophs and mouse ACTH-secreting tumoral pituitary cell line AtT-20 (Hofland *et al.* 2005, van der Hoek *et al.* 2005). In agreement, dexamethasone reduced SSTR2, but not SSTR5, mRNA and protein expression (van der Hoek *et al.* 2005, Gatto *et al.* 2020). *In vitro* experiments in primary cultured cells from ACTH-secreting PitNETs and AtT-20 cells showed higher potency of pasireotide compared to octreotide in reducing ACTH secretion (Hofland *et al.* 2005, Ben-Shlomo *et al.* 2009, van der Pas *et al.* 2013), cell proliferation by ERK1/2 inhibition (Treppiedi *et al.* 2019), and in promoting cell apoptosis (Treppiedi *et al.* 2019).

However, pasireotide treatment induces biochemical remission only in a subset of patients. The largest randomized phase III study to evaluate treatment with pasireotide in CD showed normalization of urinary free cortisol in 15–26% of patients (Colao *et al.* 2012).

To date, molecular biomarkers predictive of patients’ responsiveness to pasireotide are still under investigation. A direct correlation of the *in vivo* efficacy of pasireotide treatment with the expression of SSTR5 has not yet been proven. *In vitro* pasireotide resistance was also observed in the presence of SSTR5 (Ibáñez-Costa *et al.* 2016), suggesting that resistance can involve post-receptor mechanisms.

In the last years, some molecular factors playing a role in the modulation of SSTR5 expression and activity in ACTH-secreting PitNET cells have been identified. In particular, the cytoskeleton actin-binding protein filamin A (FLNA) and the mutational status of ubiquitin-specific peptidase 8 (USP8) have been shown to regulate SSTR5 in corticotroph tumors, suggesting clinical implications in the pharmacological responsiveness to pasireotide treatment.

## Role of cytoskeleton protein FLNA

FLNA is a large, ubiquitously expressed actin filament crosslinking protein. Due to its ability to bind a variety of partner proteins, FLNA acts as a molecular platform that orchestrates a variety of intracellular processes, including cell adhesion, migration, maintenance of cell shape, differentiation, proliferation, and transcription. Mounting evidence suggests a major role of FLNA in signal transduction, through its interaction with intracellular signaling molecules, kinases, and transcription factors (Stossel *et al.* 2001, Nakamura *et al.* 2011).

It is well known that the actin-binding protein FLNA is critically involved in the regulation of SSTR2, as well as dopamine receptor type 2 (DRD2) in growth hormone (GH)- and PRL-secreting PitNETs, respectively. FLNA modulates different steps, including expression at the cell surface, internalization, post-endocytic fate determination, and coupling with intracellular effectors, that together contribute to proper signal transduction by SSTR2 and DRD2 in pituitary tumoral cells (Peverelli *et al.* 2012, 2014, Treppiedi *et al.* 2018, 2020).

**Figure 1:**
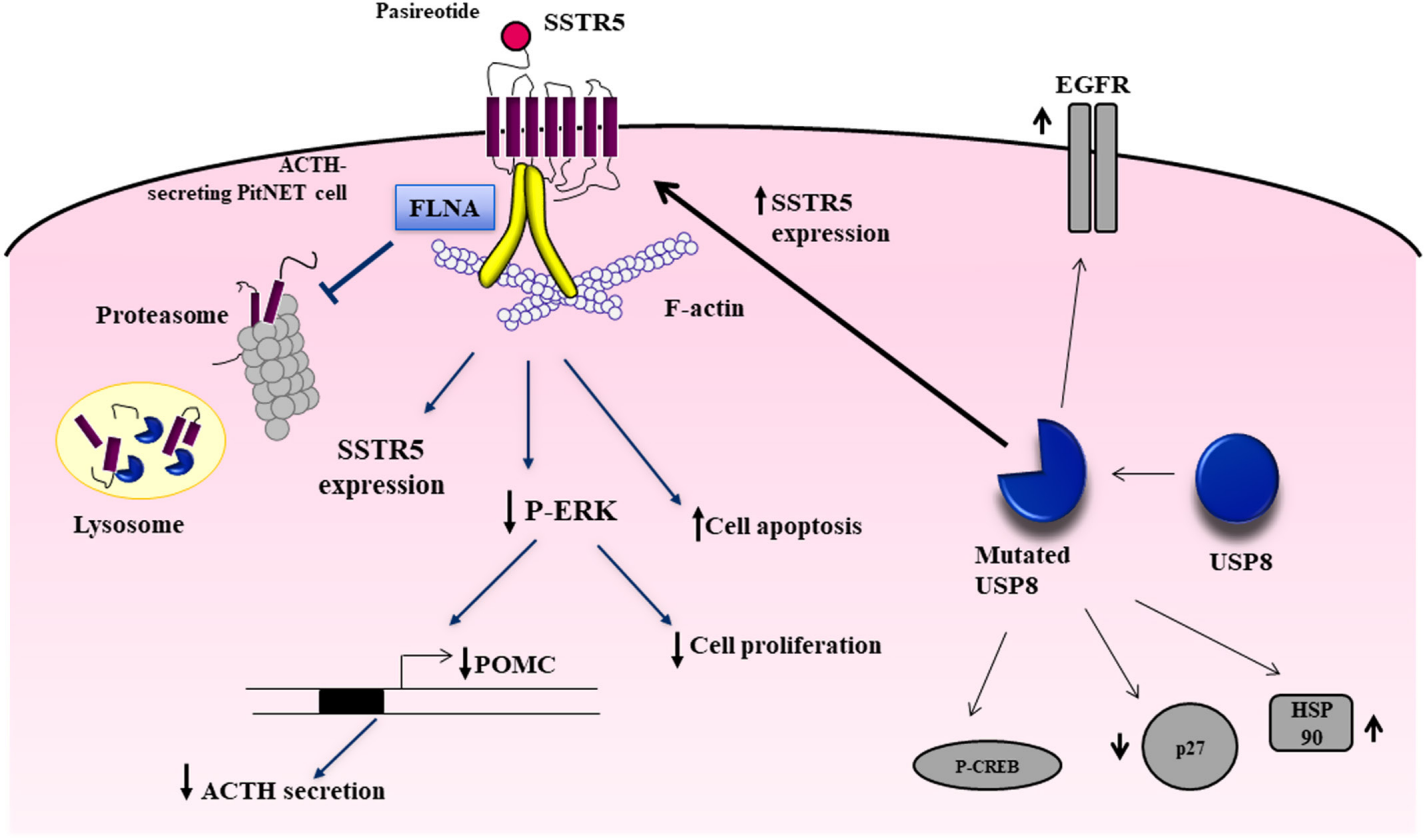
Schematic representation of FLNA and USP8 involvement in SSTR5 regulation in ACTH-secreting PitNET cells. FLNA directly interacts with SSTR5, and the amount of FLNA-SSTR5 complexes increased after pasireotide incubation. FLNA is required to maintain a stable amount of SSTR5 in basal condition by preventing both lysosomal and proteosomal degradation. FLNA is required for all pasireotide-mediated intracellular responses, including ERK1/2 phosphorylation inhibition, POMC expression and ACTH secretion reduction, antiproliferative, and proapoptotic effects. Mutations in USP8 gene have been correlated with higher levels of SST5 expression, suggesting that SSTR5 is positively regulated by mutant USP8, but the molecular mechanisms remained unknown. The targets of USP8 identified so far (EGFR, p27, HSP90, CREB) are shown.

In ACTH-secreting PitNETs, FLNA is involved in the regulation of the expression and signal transduction of SSTR5 (Fig. 1). In primary cultured cells of human tumoral corticotrophs and in AtT-20 cells, the specific silencing of FLNA induced a reduction of SSTR5 expression, suggesting that FLNA is required to maintain a stable amount of SSTR5 in basal condition by preventing both lysosomal and proteosomal degradation (Treppiedi *et al.* 2021*a*). In addition, FLNA seems to play a role in SSTR5 downregulation induced by long-term pasireotide incubation in AtT-20 cells since in the absence of FLNA, pasireotide treatment did not affect the amount of SSTR5 (Treppiedi *et al.* 2021*a*). On the contrary, in GH-secreting pituitary tumor cells, FLNA silencing did not affect SSTR2 levels, and FLNA is required to maintain SSTR2 stability after prolonged agonist stimulation by preventing lysosomal degradation and promoting efficient internalization and recycling to the plasma membrane of endocytosed SSTR2 (Peverelli *et al.* 2014, Treppiedi *et al.* 2020).

These results are in agreement with the observation that SSTR5 and FLNA protein expression levels positively correlated in ACTH- (Treppiedi *et al.* 2021*a*) and GH-secreting PitNETs (Coelho *et al.* 2019), whereas either no correlation or correlation only in patients who were responsive to SSAs was found between SSTR2 and FLNA in GH-secreting PitNETs (Peverelli *et al.* 2014, Coelho *et al.* 2019).

Overall, these observations support the notion that FLNA differentially regulates SSTR2 and SSTR5 expression, in basal condition and upon agonist challenge. Anyway, the effect of FLNA on a specific receptor can also be cell-type dependent. Indeed, DRD2 has been found to positively correlate with FLNA in PRL- (Peverelli *et al.* 2012) and GH- (Coelho *et al.* 2019) but not in ACTH-secreting PitNETs (Sickler *et al.* 2019).

A direct interaction between FLNA and SSTR5 has been demonstrated in AtT-20 cells by proximity ligation assay (Treppiedi *et al.* 2021*a*). Moreover, the authors showed that the amount of FLNA-SSTR5 complexes was increased after pasireotide incubation. This is consistent with the role played by FLNA in pasireotide-mediated intracellular responses. Indeed, in AtT-20 cells silenced for FLNA, all the effects of pasireotide, including ERK1/2 phosphorylation inhibition, cell viability reduction, cell apotosis induction and POMC expression inhibition, were abolished (Treppiedi *et al.* 2021*a*). Similarly, in primary cultures of human tumoral corticotrophs that were *in vitro* responsive to pasireotide in terms of ACTH secretion inhibition, FLNA silencing prevented the antisecretory effects of pasireotide (Treppiedi *et al.* 2021*a*).

Interestingly, the overexpression of FLNA by transient transfection did not induce significant changes in SSTR5 protein expression or in pasireotide-mediated effects, probably due to the dilution of the partner proteins involved in the FLNA-regulated processes, in line with previous observation on scaffold proteins (Burack & Shaw 2000).

Together, these findings suggest a contribution for FLNA in determining ACTH-secreting PitNET responsiveness to pasireotide. Further studies in a large cohort of patients are required to validate FLNA as marker predicting the success of pasireotide treatment of ACTH-secreting PitNETs. These studies should be taken into account that the activity of FLNA as scaffold protein is tightly regulated by several mechanisms, including FLNA phosphorylation and alternative splicing. In particular, in PitNET cells, PKA-mediated FLNA phosphorylation at Ser2152 switches FLNA function from a scaffold that allows SSTR2 and DRD2 signal transduction to a signal termination protein that hampers all SSTR2 and DRD2 antitumoral effects (Peverelli *et al.* 2018, Mangili *et al.* 2020). The impact of FLNA phosphorylation on SSTR5 expression and activity in tumoral corticotrophs, as well as the phosphorylation status of FLNA in ACTH-secreting PitNETs, remains to be determined. The expression of splice variants of FLNA has also to be considered in studies analyzing FLNA mRNA expression since FLNA splice variant-1, that lacks the domain of interaction with SSTRs and DRD2 and the Ser2152 target of phosphorylation (Pentikäinen *et al.* 2011), might interfere with full-length FLNA function.

## Role of USP8 mutational status

Gain-of-function somatic mutations in the *USP8* gene have been identified in 11–60% of patients with ACTH-secreting PitNETs (Ma *et al.* 2015, Perez-Rivas *et al.* 2015, Reincke *et al.* 2015, Hayashi *et al.* 2016, Albani *et al.* 2018, Losa *et al.* 2019, Castellnou *et al.* 2020, Sesta *et al.* 2020, Treppiedi *et al.* 2021*b*). They are missense variants or small single codon deletions that mostly occur within or in proximity to the consensus 14-3-3 binding motif RSYSSP at amino acid positions 715–720 of USP8 (Centorrino *et al.* 2018, Dufner & Knobeloch 2019), resulting in compromised 14-3-3 protein binding or more rarely in the autoinhibitory domain of USP8 (Kakihara *et al.* 2021, Treppiedi *et al.* 2021*b*), leading in both cases to constitutively enhanced deubiquitinase activity. As a consequence, the EGF receptor (EGFR), that is a well-studied target of USP8, undergoes an increased deubiquitination and recycling on the plasma membrane, and its sustained signaling contributes to cell proliferation, POMC transcription, and ACTH secretion in USP8-mutated tumoral corticotrophs cells (Ma *et al.* 2015, Reincke *et al.* 2015, Treppiedi *et al.* 2021*b*). However, further studies comparing gene and protein expression in USP8 mutated vs WT tumors revealed that USP8 mutations have pleiotropic effects, not limited to EGFR (Fig. 1). USP8 mutations affect expression levels of genes involved in different pathways, including processes related to cell-cell adhesion, cadherin signaling, Wnt signaling, and cell proliferation (Bujko *et al.* 2019). By immunohistochemistry analysis, the cell-cycle inhibitor p27 was found to be significantly reduced, whereas the chaperone HSP90 and the phosphorylation of the transcription factor CREB were increased in USP8 mutated tumors (Weigand *et al.* 2019). Microarray analysis revealed that in USP8-mutated tumors several genes associated with endosomal protein degradation and membrane components were downregulated (Sesta *et al.* 2020), and alterations in ubiquitination processes are reflected by a differential microRNA expression (Bujko *et al.* 2021).

Several studies have investigated the clinical features of USP8-mutated ACTH-secreting PitNETs, but although the mutated tumors have been described as small in size, with high ACTH secretion, and mostly found in women, the prognosis correlated with the mutational status of USP8 is still controversial, as USP8-mutated tumors seem to be associated with a greater probability of surgical remission (Losa *et al.* 2019, Castellnou *et al.* 2020) as well as a higher risk of recurrence of CD (Albani *et al.* 2018, Treppiedi *et al.* 2021*b*) compared to WT tumors.

A possible different responsiveness to pasireotide of USP8-mutated tumors has been hypothesized on the basis of the observation that SSTR5 is expressed at higher levels in mutated tumors compared to WT ones (Fig. 1). Hayashi and coauthors compared the expression of SSTR2 and SSTR5 in mutant vs WT USP8 ACTH-secreting PitNETs (Hayashi *et al.* 2016). As expected, they found that the mRNA and protein expression levels of SSTR2 were low in the entire cohort, with no differences between mutated and WT tumors. On the contrary, the mRNA expression of SSTR5 was significantly higher in USP8 mutants than in WT, with a positive correlation with USP8 mRNA. Moreover, immunohistochemistry analysis showed that SSTR5 protein expression levels were significantly higher in the mutated than in WT tumors, as well. WT and mutant tumors showed similar membrane expression patterns of SSTR5. The stepwise regression analysis identified the USP8 mutation status and USP8 mRNA expression as contributors to the SSTR5 transcript and protein expression.

These findings have been confirmed by another study that analyzed SSTR5 expression by immunohistochemistry and showed that USP8-mutant corticotroph tumors were more often SSTR5 positive and had a higher SSTR5 immunoreactivity score compared to USP8 WT tumors (Castellnou *et al.* 2020).

Although both these works suggest that SSTR5 is positively regulated by mutant USP8, the molecular mechanisms involved remained unknown. Moreover, the value of USP8 mutational status, as well as of the SSTR5 expression levels, in predicting pasireotide responsiveness in corticotroph tumors have yet to be proven.

## Other factors possibly involved in SSTRs regulation glucocorticoid-mediated β-arrestins modulation

β-Arrestin 1 and 2 are intracellular adaptor proteins that play a critical role in the desensitization, internalization, and signal transduction of GPCRs. They are recruited to the plasma membrane by ligand-activated and phosphorylated receptors, leading to G protein-mediated signal termination, receptor endocytosis by targeting to clathrin-coated pits, as well as to the beginning of specific arrestin-mediated signal transduction pathways.

The five subtypes of SSTRs differ in their patterns of β-arrestin mobilization and endosomal sorting. In particular, SSTR2 strongly and stably interacted with both β-arrestin 1 and 2, whereas SSTR5 transiently recruited only β-arrestin 2 (Tulipano *et al.* 2004, Peverelli *et al.* 2008, Treppiedi *et al.* 2020). The interaction with β-arrestin 2 has a crucial role in the regulation of SSTR5 in GH-secreting PitNETs (Peverelli *et al.* 2009, Peverelli *et al.* 2013). Importantly, in GH-secreting PitNETs, the differential expression of β-arrestins was found to be associated with the tumor responsiveness to the treatment with octreotide (Gatto *et al.* 2013, 2016).

β-Arrestins are expressed in corticotroph tumour cells as well, but no data are available on their role in the regulation of SSTRs in ACTH-secreting PitNETs, and on the correlation with medical treatment responsiveness.

It has been demonstrated that β-arrestins are differentially modulated by glucocorticoids in different cell models. In particular, glucorticoid exposure increased β-arrestin 1 and decreased β-arrestin 2, by affecting gene transcription (Oakley *et al.* 2012). The same effects were observed in AtT-20 cells (Gatto *et al.* 2020). Dexamethasone treatment of AtT-20 cells resulted in a time-dependent increase of β-arrestin 1 expression and a concomitant decrease in β-arrestin 2 at both mRNA and protein levels. These effects were abolished by a glucocorticoid receptor antagonist. The reduction of β-arrestin 2 after dexamethasone treatment might explain the increased efficacy of an SSTR5 agonist in reducing ACTH secretion from AtT-20 cells after dexamethasone pretreatment (van der Hoek *et al.* 2005).

The different exposure to glucocorticoids of an ACTH-secreting tumor, that can change during the patient clinical management, might thus affect β-arrestins expression and consequently SSTR5 desensitization, endocytosis and signaling, affecting the responsiveness to pasireotide.

## Conclusions

The multiple molecular factors that modulate the expression and function of SSTRs in ACTH-secreting PitNETs have been only partially elucidated. Although the effects of glucocorticoids on SSTR2 downregulation have been well documented, few data are available about SSTR5 regulation.

The efficacy of the pharmacological therapy with pasireotide of ACTH-secreting PitNETs might depend on several molecular factors, acting at different steps from the binding to SSTR5 at the cell surface to the activation of complex intracellular signal transduction cascades, that converge to ACTH secretion inhibition and cell growth arrest.

The cytoskeleton actin binding protein FLNA is required for SSTR5 expression and signaling, and mutations in *USP8* gene have been associated with increased SSTR5 expression, but the consequences on SSTR5 function and pasireotide effectiveness have yet to be proven. Possible effects of glucocorticoids on SSTRs desensitization, internalization, and signaling through the modulation of β-arrestins expression require further investigation.

Future research focused on the molecular mechanisms regulating SSTRs in ACTH-secreting PitNETs could provide the basis for the development of both novel biomarkers predicting drug response and novel pharmacological targets.

## Declaration of interest

The authors declare that there is no conflict of interest that could be perceived as prejudicing the impartiality of the research reported.

## Funding

This work was supported by AIRC (Associazione Italiana Ricerca Cancro) grant to G M (IG 2017-20594), Italian Ministry of Health grant to G M (PE-2016-02361797), Ricerca Corrente Funds from the Italian Ministry of Health and Progetti di Ricerca di Interesse Nazionale (PRIN) grant to E P (2017N8CK4K).
